# Nurses’ perspectives on implementing sleep protection for premature infants in the neonatal intensive care unit: a qualitative study

**DOI:** 10.1186/s12913-025-12511-4

**Published:** 2025-03-06

**Authors:** Yujing Gu, Yunfei Tang, Yan Xue, Juan Wu, Jun Xie

**Affiliations:** 1 Nursing Department, Affiliated Children’s Hospital of Jiangnan University (Wuxi Children’s Hospital), Jiangsu Wuxi, China; 2 Neonatology Department, Affiliated Children’s Hospital of Jiangnan University (Wuxi Children’s Hospital), Jiangsu Wuxi, China; 3 Department of Pediatrics, Yixing Traditional Chinese Medicine Hospital, Wuxi, Jiangsu China

**Keywords:** Neonatal intensive care unit, Sleep protection, Nurse, Premature infants, Qualitative research

## Abstract

**Background:**

The normal development of sleep-wake cycles is crucial for the long-term neurological health of preterm infants, yet sleep protection practices remain suboptimal. Given China’s large population of preterm infants and its distinct cultural background and healthcare policies compared to Western countries, NICU nurses in China may face unique challenges. However, our understanding of the barriers and facilitators encountered by nurses in implementing sleep protection for preterm infants is limited.

**Methods:**

From November 2023 to February 2024, we conducted semi-structured interviews with 15 nurses at a tertiary children’s hospital in China. The interview guide was based on the Capability, Opportunity, Motivation, and Behavior (COM-B) model. Data analysis employed inductive thematic analysis, aligning the findings with the COM-B model and the Theoretical Domains Framework (TDF) to identify key barriers to effective management and potential interventions.

**Results:**

Three major themes emerged: (1) Capability: Lack of knowledge among practitioners; Limited communication with parents; Inability to understand the cues of the baby; and Developing guidelines to support decision-making; (2) Opportunity: Time constraints; Poor interdisciplinary collaboration; Lower priority for sleep; and Additional medical expenses; (3) Motivation: Positive management attitude; Organizational expectations and support; Professional sense of responsibility; and Being an example to peers.

**Conclusion:**

To improve sleep protection for preterm infants in NICU settings, it is imperative to address several key barriers. Firstly, elevating the priority of sleep protection is essential. Specifically, a comprehensive strategy focusing on enhancing healthcare professionals’ knowledge and skills, promoting multidisciplinary collaboration, strengthening communication with parents, and optimizing human resource allocation is crucial for effectively implementing sleep protection measures.

**Trial registration:**

Not required.

## Introduction

According to the latest data released by the World Health Organization (WHO), the global preterm birth rate is approximately 9.9%, with around 13.4 million infants facing premature birth each year [1]. In China alone, the annual number of premature births is approximately 1.09 million, ranking second highest in the world [2]. Despite significant advancements in intensive care technology over the past decade, the complex complications associated with premature births (including survival, health, and neurodevelopment) continue to pose a serious public health challenge [3]. Premature infants, who are born before their due dates, may face the risk of mortality and require transfer to a well-equipped Neonatal Intensive Care Unit (NICU) for further life support. Negative environmental stimuli, such as noise, light, and non-centralized nursing procedures, disrupt the establishment of their normal sleep patterns [4, 5]. Sleep is a fundamental physiological need for humans. Evidence indicates that it plays a crucial role in brain development, closely linked to synaptic remodeling processes and impacting the development of sensory-motor exploration abilities. Sleep deprivation significantly increases the incidence of neurodevelopmental deficits in preterm infants compared to full-term newborns [6]. Furthermore, the negative effects of neurodevelopmental defects may persist into adulthood [7].

The National Association of Neonatal Nurses (NANN) proposed a sleep protection strategy in 2011, which centered on sleep-wake states to safeguard the complete sleep cycle of preterm infants [8]. The Victorian Department of Health updated its guidelines for neonatal developmental care practices in July 2020, also listing sleep protection as a goal for infant developmental care [9]. Consequently, the academic community has increasingly emphasized the role of sleep in early infant growth and development, conducting extensive research to improve sleep quality and prevent neurological complications in preterm infants [5, 10]. However, clinical implementation has been suboptimal [11–14]. One study revealed that only 48% of infants had sufficient time to complete a full 60-minute sleep-wake cycle between two clinical nursing interventions [15]. A survey of eight NICUs in South Korea found that 51.8% of nurses had never received education on neonatal developmental care [16]. In China, the emphasis on sleep management for preterm infants appears even weaker, with limited research reports available [11].

Simultaneously, Chinese nurses may face unique challenges influenced by NICU policies and traditional family culture. Integrated evidence suggests that early maternal-infant contact facilitates deeper sleep in preterm infants [17, 18]. WHO strongly recommends incorporating family involvement into routine care for preterm infants [3]. However, most NICUs in China operate as closed wards, where parents are only allowed to see their infants shortly before discharge, with communication limited to phone consultations [19]. On the other hand, influenced by cultural norms, Chinese postpartum women undergo a confinement period (zuò yuè zi) for nearly a month following childbirth. During this time, young women typically adhere to the advice of authoritative female elders (such as mothers, mothers-in-law, and grandmothers), who are regarded as experienced guides. Childbirth is perceived by older generations as a significant physical trauma that damages vital energy, making going outside a taboo [20]. This inevitably restricts certain maternal-infant interactions. Consequently, healthcare providers in China, serving as the primary caregivers and substitutes for parents during the hospitalization of premature infants, play a crucial role in safeguarding their sleep [21]. Among the healthcare professionals, nurses are the sole staff in the NICU who can provide uninterrupted care for premature infants around the clock. Proactively seeking in-depth insights from nurses can greatly enhance our understanding of their behavior.

In this study, we utilized the Capability-Motivation-Opportunity for Behavior Change model (COM-B) and the Theoretical Domains Framework (TDF) as our theoretical frameworks to comprehensively analyze the facilitators and barriers that individuals encounter during the process of behavior change [22, 23]. The COM-B model emphasizes the need to stimulate motivation (M), enhance capability (C), and provide sufficient material and social opportunities (O) in order to elicit the desired behavior (B) and help individuals achieve specific behavioral goals [22]. Capability refers to the physical and psychological capacity of individuals to change behavior, including skills, memory, and understanding. Motivation is linked to the brain processes that motivate and guide behavior change, such as planning, evaluation, and emotional responses. Opportunity entails creating environmental conditions for behavior execution, encompassing social and material factors. However, this model has certain limitations as it is overly generalized and lacks specific details. To address this, the TDF offers a solution by aligning the components of the COM-B model with its 14 domains, allowing for a focused analysis of barriers across different domains and complementing the research findings [23]. This approach has been used in studies aimed at developing interventions to effectively manage sleep disorders in brain tumor patients [24], demonstrating the usefulness of combining COM-B and TDF as an analytical framework to identify barriers and facilitators faced by healthcare providers in implementing sleep protection for premature infants, and providing potential intervention strategies to enhance their capability.

## Methods

This study was a descriptive qualitative research that adheres to the philosophical principles of “natural inquiry” with the aim of facilitating the description and exploration of phenomena and issues. Researchers can employ this method to examine a wide range of topics related to people’s experiences or perspectives, extracting valuable information concerning reactions, thoughts, driving forces, or inhibiting factors related to events [25]. By utilizing everyday language to directly describe and delve into the views of NICU healthcare professionals regarding sleep protection for premature infants, meaningful themes can be derived to obtain more authentic and unbiased primary findings. This study was conducted in accordance with the Helsinki Declaration and received approval from the Ethics Committee of the Affiliated Children’s Hospital of Jiangnan University on November 20, 2023 (Approval No. WXCH2023-11–088).

### Sampling

This study was conducted in the NICU of a tertiary children’s hospital in Wuxi, China, from November 2023 to February 2024. Purposeful sampling was employed to select nurses as participants. Purposeful sampling plays a pivotal role in qualitative research, aiming to select cases that offer profound insights, ensuring efficient research within limited resources [26]. Participants were chosen based on two criteria: they currently work in the neonatal intensive care unit with extensive clinical experience, and they were willing and actively engaged in the study. The first author (YG) contacted eligible participants via telephone, elaborating on the study’s purpose and extending invitations. The sample size was determined based on Francis et al.‘s principle of theoretical interview saturation, ensuring interviews continued until data saturation was achieved [27]. Upon completing 12 interviews, data saturation was achieved. To further validate that no new information or results emerged, an additional 3 interviews were conducted. Consequently, interview data from a total of 15 participants were included.

### Data collection

This study employed face-to-face semi-structured interviews for data collection. To ensure a quiet environment, interviews were conducted in the duty room. Appointments were made with participants in advance for one-on-one interviews. Prior to the interviews, participants were informed of the study’s purpose and data collection procedures, asked if they were willing to participate voluntarily, obtained consent, signed informed consent forms, and then the interviews were audio-recorded. The interview guide was constructed based on the COM-B model, which emphasizes the importance of capability, opportunity, and motivation in any behavior change, with these elements influencing each other and working together [22]. The COM-B model provides a comprehensive framework for designing plans aimed at promoting behavior change (Table 1). Initially, open-ended questions were used to stimulate discussion, such as “What confusions do you have during the process of sleep protection for preterm infants?” Based on their responses, more probing questions were asked, such as “How do you perceive this issue?” and “What do you think could be a solution?” Finally, participants were encouraged to provide specific examples from their daily work, such as “How is this handled in your unit?” The duration of the interviews was determined by the participants’ willingness to share, typically around 30 min. No other individuals were present during the interviews, and none of the participants withdrew from the study. To ensure privacy, participants were identified by a code (N1 ~ N15) instead of their names.

**Table 1:** Outline of the interview

COM-B	COM-B domains	Interview Questions
Capability	Knowledge	- What is your level of understanding regarding the role of sleep in premature infants? - What particular perplexities or uncertainties arise during your endeavor to safeguard the sleep of premature infants?
	Skills	- What skills do you think you need to possess in sleep protection for premature infants?
Opportunity	Social opportunity	- What effects did the parents of premature infants have on your preterm sleep protection? - How do your colleagues influence your sleep protection for premature infants?
	Physical opportunity	- What support do you think is needed for sleep protection in premature infants? - What support do you think your organization has provided you with?
Motivation	Reflective motivation	- What factors affect your willingness to provide sleep protection for premature infants? - What are your expectations for implementing sleep protection for premature infants in your department?
	Automatic motivation	- What do you think is needed to motivate you to provide sleep protection for premature infants?

### Rigour and reflexivity

First, the interviews were conducted by the first author, YG, who had no NICU experience and was pursuing a master’s degree in pediatric nursing, thereby mitigating potential biases associated with professional identity. Second, to enhance the reliability of the findings, we implemented a member checking process, allowing all participants to review and modify their responses after the interviews. Third, to improve the confirmability of the results, the researcher endeavored to maintain objectivity during data collection and analysis, avoiding the influence of personal hypotheses. Finally, the generated themes were verified with the participants to ensure they accurately reflected the participants’ experiences.

### Data analysis

In this study, we employed a hybrid analytical approach, integrating inductive thematic analysis with deductive analysis guided by the TDF framework [28, 29]. This method enabled us to extract salient themes directly from the data and connect them to the COM-B model and TDF domains for deeper interpretation. Initially, YG and YX meticulously proofread the transcribed text to ensure a thorough understanding of the data. Subsequently, they identified and coded key factors influencing the implementation of sleep protection measures for preterm infants, categorizing these factors into distinct coding labels to lay the groundwork for preliminary classification. Building on this, YG and YT further explored the connections between these factors and the theoretical frameworks. For recurring themes, YG and YT engaged in in-depth discussions to determine the final themes. Subsequently, YG and JX deductively mapped these themes onto the theoretical domain framework, carefully considering the specific definitions of each component. Throughout the coding and mapping process, all authors resolved any discrepancies through collective discussions, ensuring the consistency and accuracy of the study. We utilized Microsoft Excel for data organization, with all other coding and categorization tasks being manually executed. The comprehensive criteria for reporting qualitative research (COREQ) were adhered to [30].

As our participants communicated in Chinese, to mitigate potential translation errors due to language differences, the following steps were implemented: The initial translation of the manuscript was conducted by author YG, who passed the university’s English writing course. The corresponding author, JX (currently pursuing a medical doctorate), performed quality control on the translation and finalized the English version.

## Results

### Characteristics of participants

A total of 15 nurses participated in the interviews, comprising one male and fourteen females. Their ages ranged from 22 to 46 years, with an average age of 33.7 years. Their work experience varied from 2 to 27 years. The demographic characteristics of the participants are presented in Table 2.

**Table 2:** Demographic characteristics (*n* = 15)

	*N*	%
Sex		
Male	1	6.7
Female	14	93.3
Age		
20–30 years	5	33.3
31 − 40 years	8	53.3
41 − 50 years	2	13.3
Work experience		
1–5 years	4	26.7
6–10 years	3	20.0
11–15 years	3	20.0
16–20 years	4	26.7
> 20 years	1	6.7

### Key findings

**Fig. 1:**
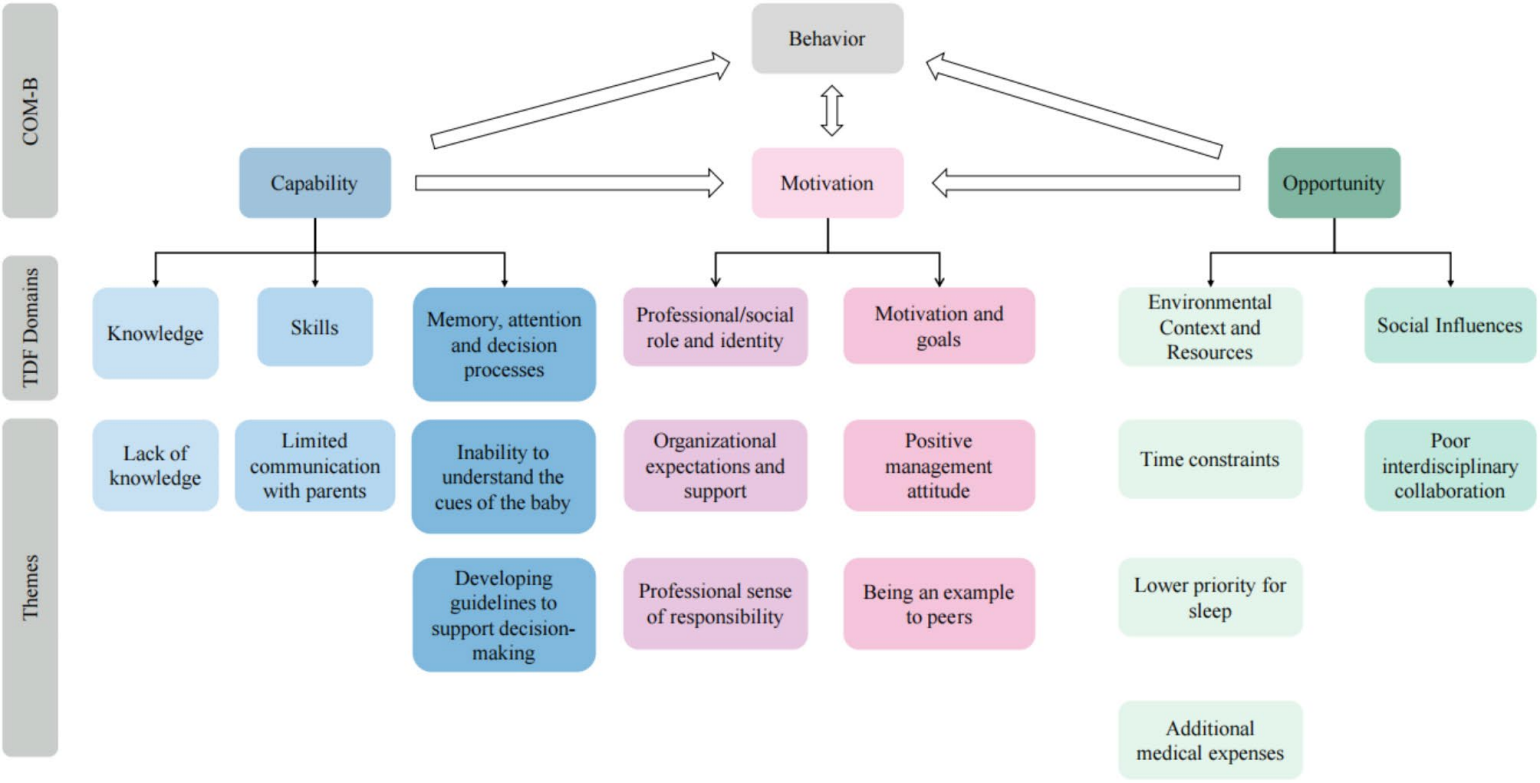
Mapping of themes to the COM-B Model

The identified themes were mapped onto the theoretical framework, as illustrated in Fig. 1.

### Theme 1: capability

#### Lack of knowledge among practitioners

The majority of interviewees highlighted the crucial role of sleep in the growth and development of premature infants. However, there is currently insufficient knowledge about sleep in premature infants. Identifying the different sleep stages in premature infants was considered a primary challenge by the participants. This skill primarily relies on the clinical experience of nurses. One nurse stated:

*"We currently differentiate the sleep state of premature infants mainly based on experience. For example, if a baby is in deep sleep, they generally won't wake up even if we make noise. But sometimes, we observe their eyeballs moving, indicating light sleep or being awake."(N4)*

Almost all nurses emphasized the lack of specialized education resources on premature infant sleep and believed that receiving training and education was a prerequisite for promoting sleep protection in premature infants. Nurses also emphasized the importance of developing skills in managing premature infant sleep through daily practice and on-the-job learning, especially in bedside care. Additionally, they expressed a desire for guidance from senior colleagues. For instance, one interviewee said:

*"Sleep protection? Currently, we don't have (such educational resources). We have the concept, but we haven't received systematic learning." (N1)*

*"What we really hope for is the integration of theory and practice, such as real-time bedside teaching during work. It would be best if experienced nurses could provide appropriate guidance." (N4)*

#### Limited communication with parents

All nurses highlighted the evident benefits of parental involvement in the sleep protection of premature infants. Providing sleep education to parents and involving them actively can effectively enhance the quality of sleep for premature infants. However, due to certain constraints, such as closed ward management and limited parental energy, there is insufficient communication. This can result in a non-interactive relationship between parents and staff. As one nurse expressed:

*“I am aware that mother-infant contact can promote sleep*,* but*,* you know… in the intensive care unit environment*,* it is challenging to achieve. Our communication with parents relies solely on phone calls.” (N1)*.

#### Inability to understand the cues of the baby

Some interviewees mentioned that they couldn’t provide sufficient cues due to the uniqueness of premature infants. Nurses often have to wake up sleeping babies following their workflow and perform feeding tasks every two hours. Despite raising the question of “how to balance sleep and feeding” (N5), nobody attempted to explore it. Even though parents have raised this concern multiple times, nurses often lack confidence in their responses. As one nurse stated:

*“At discharge*,* some parents ask if we need to wake up the child for feeding and diaper changes. At that moment*,* I don’t know how to answer because I’m unsure myself.” (N15)*.

#### Developing guidelines to support decision-making

Certain interviewees mentioned their insights into facilitating sleep among premature infants, albeit with some reservations. On one hand, they expressed a longing to offer various forms of support, such as the utilization of music therapy. On the other hand, due to the absence of dedicated guidelines catering to sleep support in preterm infants, certain experiences are left unexplained or unsubstantiated. They expressed a yearning for the formulation of comprehensive directives to bolster clinical decision-making, as evidenced by the following quotation:

*“In our clinical encounters*,* we have come across peculiar phenomena*,* such as some children exhibiting a preference for gentle melodies while others seem to gravitate towards rock music. Naturally*,* we exercise control over the volume of the rock music. However*,* presently*,* there appears to be an absence of empirical evidence substantiating the provision of rock music to them.” (N11)*.

### Theme 2: opportunity

#### Time constraints

The majority of interviewees opined that the implementation of sleep management for premature infants is constrained by factors such as the severity of the illness, time limitations, and manpower resources. The demanding workload leads to limited attention given to the sleep condition of premature infants. Another frequently raised issue is the inability to promptly respond to a crying premature infant. As illustrated in the following extract:

*“Sometimes we are too busy. When a baby is crying and we are feeding other babies*,* do we need to stop and comfort the crying baby? Although we know that his crying may affect other sleeping babies.” (N14)*.

#### Poor interdisciplinary collaboration

Different teams, such as nurses, doctors, and medical imaging specialists, to varying degrees, disrupt the sleep of premature infants on a daily basis. Due to a lack of communication among team members, medical rounds are often repetitive and irregular, and scattered instructions negatively impact the sleep of premature infants. Despite nurses repeatedly emphasizing “shared responsibility” (N5, N10, N14). For example, a nurse expressed:

*“At times*,* doctors issue instructions intermittently*,* especially for invasive procedures. We do hope for consolidated actions*,* but sometimes doctors remember*,* ‘Oh*,* I forgot to order a blood glucose test.’ Then later*,* ‘Oops*,* I forgot to order an arterial blood gas test.’” (N11)*.

#### Lower priority for sleep

The priority given to protecting the sleep of premature infants is relatively low. Respondents believe that addressing known health threats, including feeding, takes precedence. Another frequently mentioned circumstance is that interventions tend to be scattered when premature infants face urgent situations requiring resuscitation, with certain principles likely being compromised. For example, the following statement:

*“When a baby’s condition changes*,* we don’t pay attention to any sounds. Everything else becomes secondary; the focus is on rescuing them…” (N6)*.

Furthermore, several respondents mentioned the delicate balance between risks and benefits. The compromise of sleep is sometimes seen as an inevitable consequence of using certain medical devices such as monitors and incubators. However, solely shutting down these machinery for the sake of sleep entails significant risks. As one nurse aptly expressed:

*“The sounds of the monitor cannot be eliminated. Once silenced*,* I would be undertaking tremendous risks…” (N7)*.

#### Additional medical expenses

Some interviewees believe that cost is a major obstacle when considering the need for monitoring the sleep of premature infants. On one hand, there are concerns about additional medical expenses if objective assessment tools, such as electroencephalography (EEG), are employed. On the other hand, using subjective rating scales implies that nurses would shoulder “extra unpaid responsibilities” (N15). As illustrated in the following quotes:

*“…we don’t have the capacity to evaluate how well a baby is sleeping. If sleep assessment becomes part of routine care*,* it would add a significant workload.” (N8)*.

*“Using EEG for sleep monitoring inevitably entails substantial additional costs. It’s very challenging…because not everyone can afford it.” (N1)*.

### Theme 3: motivation

#### Positive management attitude

Through the interviews, we discovered that the majority of nurses subconsciously recognize the significant necessity of sleep management for premature infants. It not only pertains to the developmental growth of the infants themselves but also holds equal importance for nursing safety and personal competence enhancement. For instance, consider the following statements:

*“I believe that implementing sleep management is necessary. Because in the future*,* I’ll have children of my own*,* and learning how to manage their sleep will be helpful to myself as well.” (N7)*.

*“Undoubtedly*,* administering sleep management for premature infants is beneficial. Because sleep-related sudden death can easily occur in preterm infants*,* it is imperative to conduct sleep monitoring for such high-risk babies.” (N9)*.

#### Organizational expectations and support

Organizational culture refers to the shared values, beliefs, and behavioral guidelines among members of an organization [31]. The support from leadership plays a crucial role in fostering continuous, proactive knowledge acquisition, and quality control. The majority of respondents emphasized the importance of support and assurance from managerial levels. For example, one nurse stated:

*“Our leaders frequently conduct quality control on our nursing work. This includes knowledge-based questioning during our department’s morning meetings*,* which subtly motivates us to learn new knowledge and techniques.” (N12)*.

#### Professional sense of responsibility

Nurses possess a sense of ownership, and their professional sense of responsibility and desire for favorable outcomes for pediatric patients’ families serve as powerful motivating factors. This includes primarily enhancing the quality of nursing services and/or ambitiously translating research findings into practice.

*“In our day-to-day processes*,* we have already made efforts…for instance*,* lowering our voices. It’s necessary to inform us about the areas that require improvement.” (N1)*.

*“…Additionally*,* we can compile the knowledge we acquire into a booklet and distribute it to parents for their learning when premature infants are discharged.” (N5)*.

#### Being an example to peers

Some nursing personnel believe that obtaining recognition from colleagues and having the opportunity to share their experiences can enhance their sense of self-fulfillment. Strategies include utilizing “role modeling” to establish exemplary figures to imitate or admire. Additionally, providing appropriate financial incentives can improve behavioral competence.

*“Establishing an evaluation system that includes self-assessment and peer evaluation…outstanding managers should receive financial rewards.” (N14)*.

*“Each month*,* the department can select an outstanding premature infant sleep manager*,* which*,* over time*,* serves as a role model for the department.” (N15)*.

## Discussion

Clinical nurses, as the primary caregivers during the hospitalization of premature infants [19], play a crucial role in promoting complete sleep cycles for these infants. The focus of this study was to explore the abilities, opportunities, and motivations perceived by nursing staff when implementing an NICU premature infant sleep management program. Our findings indicate that the themes are interconnected and intertwined. All participants acknowledged the benefits of sleep protection for premature infants but unanimously recognized the pressing need to address barriers during the implementation process.

Regarding competence, our data reflect that the majority of nursing staff have insufficient mastery of knowledge and emphasize the necessity for specialized training in comprehensive premature infant sleep knowledge. Recognizing the different stages of sleep in premature infants poses a primary challenge for caregivers. Distinguishing between “active sleep” and “wakeful activity” is primarily dependent on clinical experience, which aligns with previous research findings [12]. To our knowledge, the content of sleep medicine education in medical school curricula in China is limited and disregarded. Sleep medicine has not been recognized as an independent discipline in China until recently when it was incorporated into the national specialist physician training system by the Chinese Medical Association in 2019 [32, 33]. However, positive changes are only possible after acquiring a greater understanding of premature infant sleep [34]. Therefore, we strongly recommend implementing education and training programs, organizing group discussions, and utilizing instructional resources such as videos or animations to clearly differentiate the various sleep stages of preterm infants. Additionally, feedback and evaluation components should be included to encourage continuous learning and enhance professional practice.

Meanwhile, nurses believe it is crucial to establish routines and set standards in the ward to reduce decision-making discrepancies, as consistency is paramount. For instance, different nursing staff may provide varying answers to the same question. Without specific guidance, parents feel lost in the jungle of premature infant sleep management [20]. Yet, to our knowledge, there currently seems to be no specific evidence-based guidelines for sleep in NICU premature infants [12]. Related research conducted in developed countries has formulated key guidelines based on moderate evidence and utilized quality improvement policies to promote optimal sleep management for premature infants [35], bridging the gap between evidence and practice. In other areas of premature infant care, such as pain management and breastfeeding, extensive research has been conducted [36, 37], whereas limited research exists in the domain of sleep.

Utilizing fragmented time for immediate bedside guidance is deemed highly efficient in a professional setting. Additionally, it is recommended to provide alternative avenues of learning to accommodate various learning style preferences. For instance, to strike a harmonious balance between academic pursuits and personal life, leveraging online educational platforms that transcend temporal and spatial limitations can alleviate the time burden faced by healthcare practitioners engaged in shift work [38]. As healthcare providers deepen their understanding of the physiology of infant sleep, their confidence in guiding parents also escalates [34]. It is paramount to involve family members in the care process and empower them, as the involvement of parents yields optimal intervention outcomes [39]. However, a study revealed that only 22.5% of parents received sleep education regarding their infants from healthcare providers [40]. As noted by our interviewees, within the context of China’s closed management system and the conditions necessitating postpartum confinement, nurses have limited opportunities to facilitate mother-infant contact. Nevertheless, nurses should acknowledge that fathers are assuming roles that mirror traditional maternal caregiving roles. Husbands, as fathers to the child and companions to the mothers, should shoulder the responsibility of bridging the gap between the family and the hospital [20]. Our educational sleep training aims to enable parents to detect and prevent sleep issues early on and continue implementing interventions to promote sleep even after discharge, thereby exerting efforts towards the growth and development of premature infants [41].

Regarding opportunities, currently, obstacles impede the smooth implementation of sleep management for premature infants. In this study, a majority of healthcare professionals indicated that heavy workloads, insufficient allocation of human resources, and poor interdisciplinary collaboration adversely affect the progress of sleep management for premature infants. In China, most NICUs operate in an overloaded state, and a considerable number of nursing interventions still rely on “timed care” to ensure successful execution. However, the intensity of the workload does not align with the available human resources. A NICU nurse often shoulders the responsibility of caring for three or more infants [42], undeniably impacting the availability of remaining time. This may be one of the reasons why our interviewees perceive sleep management as a lower priority. Particularly, when facing emergencies such as resuscitation, this situation becomes more pronounced. Hence, we suggest the implementation of visual aids, such as prominently placed warning signs in the ward, to enhance awareness. Simultaneously, optimizing resuscitation procedures and equipping devices like pagers can minimize sudden high-frequency noises. Additionally, offering family-centered care (empowerment) seems to be a potential approach to address the shortage of nursing staff.

The National Institute for Health and Care Excellence (NICE) in the United Kingdom recommends healthcare providers to monitor the sleep of premature infants. However, our study highlights the absence of sleep assessment as part of routine care within the healthcare system, which aligns with the findings of Bethany et al. [35]. Demanding the execution of sleep assessment under limited manpower and already strained capacities is evidently impractical. As one participant in this study described, the continuous manual evaluation of sleep is labor-intensive, implying the potential undertaking of additional unpaid responsibilities. Robinette et al.‘s project team [35] plans to collaborate with the information technology department to incorporate sleep assessment scales into the electronic health system and provide staff training on their utilization. It seems plausible to utilize remote monitoring technology and automated data analysis techniques to evaluate different sleep-wake states in premature infants. Employing a display screen to inform healthcare providers about real-time conditions of premature infants can help avoid performing care procedures during quiet sleep states, thereby preserving their complete sleep cycles [43, 44].

Poor interdisciplinary collaboration has also been mentioned in other studies [42]. Evidence indicates that the duration of interdisciplinary rounds conducted by the medical team in the NICU exceeds two hours [45]. Inconsistent workflow and inadequate communication among healthcare professionals contribute to the inefficiency of bedside rounds in the NICU, resulting in prolonged medical rounds that, in turn, induce fatigue and hinder concentration [46]. This may be one of the reasons behind the intermittent and discontinuous performance of invasive procedures. Therefore, we recommend implementing a quality improvement plan to standardize the process of medical rounds.

Regarding motivation, when healthcare providers realize the benefits of sleep for premature infants, such as promoting better growth and development, they are more driven to engage in sleep management. Studies by Hong et al. [16] demonstrate a positive correlation between healthcare providers’ attitudes toward sleep-related developmental support and the effectiveness and quality of nursing care. However, if individuals do not perceive personal benefits from a particular behavior, they may struggle to sustain it in the long term. Some interviewees in this study considered sleep management for premature infants to be mutually beneficial, including enhancing their own parenting experience and reducing the likelihood of safety hazards. Furthermore, motivation is influenced by departmental culture [47]. Organizational culture profoundly impacts the operations, development, attitudes, behaviors, and performance of its members, effectively guiding and motivating them to enhance the competitiveness of the entire organization [48]. Strong leadership support is crucial as a driving force for change. Establishing reward mechanisms and the power of role models are potential avenues to encourage professional development and increase engagement. Motivation is expected to be a dynamic process [49]. Motivation under the framework of reward mechanisms is based on individual interests, while a sense of fulfillment derived from benefiting others and recognition by peers contributes to the transformation from individual interests to a child-centered ambition (i.e., reflective motivation). In our study, reflective motivation inspired individuals to develop themselves within complex contexts and empowered them to become agents of change.

### Implications

This study demonstrates the crucial role of nurses in the protection of sleep for premature infants and proposes potential intervention measures (summarized in Table 3). Nurses can enhance “capability,” improve existing “opportunities,” and foster “motivation” to enhance the quality of care. Based on these findings, we suggest some acceptable and feasible improvements. For instance, bedside training and specialized knowledge education can enhance the capacity for sleep management. Establishing relevant standards and guidelines can help reduce decision-making dilemmas. Optimizing human resource allocation, addressing inadequate interdisciplinary collaboration, and streamlining workflow can address certain physical conditions. Lastly, implementing reward mechanisms and modeling can strengthen nurses’ motivation. Our research findings offer significant insights for developing targeted intervention strategies to optimize sleep protection for preterm infants, which may serve as a valuable reference for other countries.

**Table 3:** TDF and COM-B application matrix

COM-B components	TDF Domains	Themes	Suggestions for intervention
Capability	Knowledge	Lack of knowledge among practitioners	*Education & Training*: • Organize and carry out specialized education and training, covering topics including the special needs of premature infants for sleep, optimization of sleep environment, methods of sleep monitoring and evaluation, identification and management of sleep disorders, etc. • Combining theory and practice, through lectures, case analysis, simulation exercises, and field internships, participants are helped to deeply understand the importance of sleep protection and master effective strategies and techniques. • Invite parents to participate in sleep education classes (online and offline) to improve their skills in promoting sleep. *Environmental restructuring & Enablement*: • Developing a clinical pathway entails implementing a screening question or tool, assessment tools, and a guided list of interventions.
	Cognitive and interpersonal skills	Limited communication with parents	
	Memory, attention, and decision processes	Inability to understand the cues of the baby	
		Developing guidelines to support decision-making	
Opportunity	Environmental context and resources	Time constraints	*Environmental restructuring*: • Determining the information resources and personnel available to support sleep, organizing a sleep protection team consisting of professionals such as sleep experts, pediatricians, nurses, and experts from other relevant fields. • Implementing quality improvement projects and standardizing medical rounding processes. • Organize regular rescue drills to simulate real-life emergency scenarios, allowing healthcare professionals to familiarize themselves with emergency protocols, clarify their respective roles, and coordinate teamwork. To reduce the high-frequency noise that may occur during rescues, consider equipping pagers or other wireless communication devices. • Collaborate with the information department to develop a sleep assessment tool. • Family-involved care is a potential solution approach. *Incentivisation & Persuasion*: • Providing financial incentives/overtime subsidies to nurses appropriately. *Education*: • Organize in-house training for interdisciplinary teams to clarify team responsibilities.
		Lower priority for sleep	
		Additional medical expenses	
	Social influences	Poor interdisciplinary collaboration	
Motivation	Professional/social role and identity	Organizational expectations and support	*Incentivisation & Persuasion*: • Raise awareness among nurses about potential long-term complications associated with insufficient sleep in premature infants. • Provide financial rewards to nurses who demonstrate outstanding performance appropriately. *Establish role models to emulate or admire*: • Organize exchange meetings to share management experiences.
		Professional sense of responsibility	
	Motivation and goals	Positive management attitude	
		Being an example to peers	

### Limitations

This study has certain limitations that need to be considered. Firstly, the difficulty in gathering a sufficient number of healthcare professionals for focus group discussions at the appropriate time may have limited the richness of our data. Additionally, this study was conducted in a single hospital in China, hence the research findings may not be representative of the perspectives of healthcare professionals in other regions. Lastly, our interviews only included practicing nurses and did not encompass the viewpoints of doctors and family members. Future research should adopt a more comprehensive organizational approach to facilitate an in-depth exploration of sleep health in premature infants.

## Conclusion

In this study, we initially employed the COM-B and TDF models to ascertain the perceived capabilities, opportunities, and motivations of Chinese NICU healthcare providers in the realm of sleep management for premature infants, thereby offering novel insights to this field. The theory of behavior change asserts that both capability and opportunity influence the generation of motivation and behavior. To put it differently, the prerequisite for translating existing motivation into action lies in overcoming barriers within capabilities (e.g., knowledge, skills) and opportunities (e.g., time constraints, lack of manpower). Considering the demonstrated enthusiasm of healthcare providers towards sleep management for premature infants, our strategic focus should be dedicated to prioritizing the resolution of obstacles related to opportunities and capabilities, with the aim of fostering the utmost progress in this endeavor.

## Data Availability

The data collected and analyzed during this study are available from the corresponding author on reasonable request.

## Abbreviations

WHO: The World Health Organization
NICU: Neonatal Intensive Care Unit
NANN: The National Association of Neonatal Nurses
COM-B: The Capability-Motivation-Opportunity for Behavior Change model
TDF: Theoretical Domains Framework
TTA: Theoretical Thematic Analysis
NICE: The National Institute for Health and Care Excellence
